# 71 Burn Intensive Care Unit Early Mobility Competency Based Orientation

**DOI:** 10.1093/jbcr/irac012.074

**Published:** 2022-03-23

**Authors:** Jennie McGillicuddy, Gina Bello, Kiane Bergeron, Jeanne Lee

**Affiliations:** UC San Diego Health, Alpine, California; UC San Diego Health, San Diego, California; UC San Diego Health, Poway, California; UC San Diego Health Regional Burn Center, San Diego, California

## Abstract

**Introduction:**

Competencies in healthcare are used to teach practice standards, to establish expectations for professional growth, and to evaluate and improve the effectiveness of educational programs. Benefits of early mobilization of Burn Intensive Care Unit (BICU) patients include improved ICU related weakness and delirium, range of motion, and decreased length of stay while promoting functional independence. A rehabilitation competency was developed for early mobility with the patient in the BICU using nationally agreed-upon standards for competence. By design this competency for BICU early mobility is part of a tiered training structure, with the burn rehabilitation and pediatric competencies serving as additional tiers of burn therapist training at our organization. The goal of this competency is to provide direct therapist training, including alignment with the inter-professional team involved with this intervention.

**Methods:**

A pretest was issued regarding BICU early mobility competency. The pretest asked the therapist to indicate their perceived level of competence in the areas of functional mobility, indications and contraindications of BICU early mobility, Respiratory Care Provider (RCP) protocols and documentation of BICU early mobility. Based on the results of this pretest, it was identified that BICU early mobility would be prioritized as the next training tier. The BICU Early Mobility Competency was created in Competency Based Orientation (CBO) format, including nationally agreed-upon standards for burn therapist competence. The method of this training is designed to occur with an ongoing patient case study for validation, with the CBO as the teaching tool.

**Results:**

Creation of this competency as part of a tiered training allows our burn center to have an educational program which aligns with the nationally agreed-upon standards for burn therapist competence. Creation of this competency resulted in direct communication channels between rehabilitation, RCP, nursing and physician teams.

**Conclusions:**

This tiered system allows for time management of the validator signing off on competencies, and encourages use of current resources and literature in the areas of burn therapist competence and professional development.

